# Spectral analysis with highly collimated mini-LEDs as light sources for quantitative detection of direct bilirubin

**DOI:** 10.1186/s11671-024-03957-2

**Published:** 2024-01-18

**Authors:** Zhi Ting Ye, Shen Fu Tseng, Shang Xuan Tsou, Chun Wei Tsai

**Affiliations:** 1 Department of Mechanical Engineering, Advanced Institute of Manufacturing with High-Tech Innovations, National Chung Cheng University, 168, University Rd., Min-Hsiung, Chia-Yi, 62102 Taiwan, ROC; 2 Graduate Institute of Photonics and Optoelectronics, National Taiwan University, No. 1, Sec. 4, Roosevelt Rd., Taipei, 106319 Taiwan, ROC

## Abstract

Because the human eye cannot visually detect the results of direct bilirubin test papers accurately and quantitatively, this study proposes four different highly collimated mini light-emitting diodes (HC mini-LEDs) as light sources for detection. First, different concentrations of bilirubin were oxidized to biliverdin by FeCl_3_ on the test paper, and pictures were obtained with a smartphone. Next, the red, green, and blue (RGB) channels of the pictures were separated to average grayscale values, and their linear relationship with the direct bilirubin concentration was analyzed to detect bilirubin on the test paper noninvasively and quantitatively. The experimental results showed that when green HC mini-LEDs were used as the light sources and image analysis was performed using the G channel, for a direct bilirubin concentration range of 0.1–2 mg/dL, the G channel determination coefficient (R^2^) reached 0.9523 and limit of detection was 0.459 mg/dL. The detection method proposed herein has advantages such as rapid analysis, noninvasive detection, and digitization according to RGB grayscale changes in the images of the detection test paper.

## Introduction

Bilirubin is a yellow pigment produced by the redox reaction of hemoglobin in the red blood cells. In the human body, it normally circulates through the blood to the liver, where it combines with glucuronic acid by the UDP-glucuronosyltransferase (UGT) system. Then, it is excreted with bile into the intestine, where it is finally converted to urobilinogen and expelled via urine or feces [1, 2]. Bilirubin before and after binding with the liver cells is called as indirect or unconjugated bilirubin and direct or conjugated bilirubin, respectively, while the total bilirubin is given by the sum of indirect and direct bilirubin [3, 4]. A high indirect bilirubin index can affect brain development, which is a condition known as jaundice or hyperbilirubinemia. This is partly attributed to the inability of the liver cells to convert indirect bilirubin to direct bilirubin quickly, resulting in excessively high indirect bilirubin in the blood. If the direct bilirubin index is too high, it is clinically referred to as conjunctive or bile-retentive jaundice, and the possibility of biliary atresia should be considered in neonates [5]. Neonatal jaundice affects 60% and 80% of full-term and preterm neonates, respectively. Therefore, detection of bilirubin is a routine test item for each newborn [6, 7]. Newborns often have physiological jaundice because their liver is not fully developed, and the liver cells lack the ability to bind bilirubin. This problem disappears when the body’s ability to bind bilirubin matures [8–10]. In addition, neonates may develop pathological jaundice due to hepatitis or biliary atresia. In biliary atresia, early diagnosis and surgery are necessary to avoid the eventual need for a liver transplant [11–13]. The normal concentration range of total bilirubin in human serum is approximately 0.2–1.2 mg/dL. If the bilirubin index remains too high, it may lead to conditions such as hepatitis, athetosis, and even brain damage [14, 15]. Currently, the most common approach for detecting bilirubin in clinical medicine is the diazo method [16], which uses serum and diazo reagents in a strong acidic environment. Through the bilirubin reaction, diazo salts are combined to form dark-red azo compounds, which is then measured using a photometer to obtain the fluorescence intensity and proportion of bilirubin concentration for colorimetric determination [17–19]. However, the diazo reagents are easily affected by the pH of the environment, which can cause detection errors [20–22]. Bilirubin oxidase (BOD) is a multi-copper oxidase belonging to the class of oxidoreductases. It uses metal ions to catalyze the oxidation of bilirubin to biliverdin and has been widely employed as a reagent for the detection of bilirubin [23]. The laboratory mainly uses enzymatic methods to measure the direct and serum bilirubin concentrations. When direct bilirubin is oxidized by BOD under acidic conditions to form biliverdin, the original concentration of bilirubin is determined according to the generated bilirubin [24–27]. Although BOD has been widely used as a reagent for the detection of bilirubin, the enzymatic method still suffers from low sensitivity, high cost, and insufficient stability [28]. Fluorescence detection mainly uses metal nanodots or quantum dots to calibrate the detection object, and it uses the change in fluorescence intensity to achieve analysis [29–34]. Although the fluorescence method achieves the goal of quantitative detection of bilirubin, it has shortcomings, such as limited applications of fluorescence imaging in vivo and in vitro, and easy interference with samples containing relatively complex components so difficulty with using in clinical practice [35]. The blood test by venipuncture is currently the most accurate method for the clinical detection of human bilirubin in hospitals, but blood drawing is invasive and increases the risk of infection for special patients, time-consuming, expensive, and painful for the patients [36, 37]. Therefore, some noninvasive methods have also been proposed [38, 39]. However, with the current test-paper detection method, the change in color can only be distinguished through qualitative visual judgment by the human eye, resulting in low detection accuracy when the concentration of bilirubin in the urine is low or when the color change is not apparent. Compared with light sources used in traditional analytical instruments, the full-width at half maximum (FWHM) of light-emitting diodes (LEDs) is narrow and usually less than 30 nm, which can improve the utilization efficiency of light. Therefore, LEDs have been applied in color analyses, capillary absorption detection, photoacoustic spectroscopy, microscopic fluid control devices, and fluorescence spectroscopy [40]. Recently, smartphones have developed rapidly in terms of size, processing speed, and camera resolution, so the application of the smartphone as a high-performance image detection instrument has become possible. Moreover, the popularity of the smartphone also avoids the traditional need for complex and expensive equipment [41]. Pairing a red, green, and blue (RGB) analysis app or software with the smartphone offers advantages for developing cost-effective, portable, and easy-to-use methods that enable real-time and on-site detection of the analytes [42].

To solve the problem of the inability of the human eye to visually discriminate color changes to the test paper, this study proposes image spectral analysis with highly collimated mini-LEDs (HC mini-LEDs) as the light sources for quantitative detection of direct bilirubin. The measurement results showed that when using green HC mini-LED as the light source and using the G channel for image analysis, the G channel determination coefficient (R^2^) reached 0.9523 in the direct bilirubin concentration range of 0.1–2 mg/dL, with a detection limit (LOD) of 0.459 mg/dL. The detection method proposed in this paper has the advantages of rapid analysis, non-invasive detection, rapid detection, and quantitative analysis.

## Materials and methods

### Experimental materials

Direct bilirubin powder (Bilirubin Conjugate, Ditaurate, Disodium Salt – Calbiochem, Merck Millipore 201102, Inc., USA) is the current standard used by medical institutions to simulate direct bilirubin. The certificate of analysis (CoA) of the direct bilirubin powder was obtained according to Regulation (EC) No. 1907/2006. After using a precision scale to weigh the direct bilirubin powder, 25 mL of deionized water was added to 0.5 mg of the powder and stirred magnetically at 500 rpm for 5 min until evenly mixed to form 2 mg/dL of the solution. Then, 2 mg/dL of the direct bilirubin solution was added to deionized water and diluted into 12 standard solutions of different concentrations ranging from 0.1 to 2 mg/dL. This study used Fouchet’s reagent (S-Y Fouchet’s reagent-SY8076-1–11011019-Shih-Yung Instruments Co., Ltd., Taiwan) as the oxidant of bilirubin, and the prepared direct bilirubin solutions were oxidized with Fouchet’s reagent. In the reaction, bilirubin was oxidized and reduced to blue/green biliverdin and Fe^3+^. The bilirubin redox reaction is shown in Eq. (1).

1 $${\text{Biliverdin}} + {\text{FeCl}}_{3} \mathop{\longrightarrow}\limits^{{\left( {{\text{Fouchet}}^{\prime}{\text{s}}\;{\text{reagent}}} \right)}}{\text{Biliverdin}} + {\text{Fe}}^{3 + }$$

**Fig. 1:**
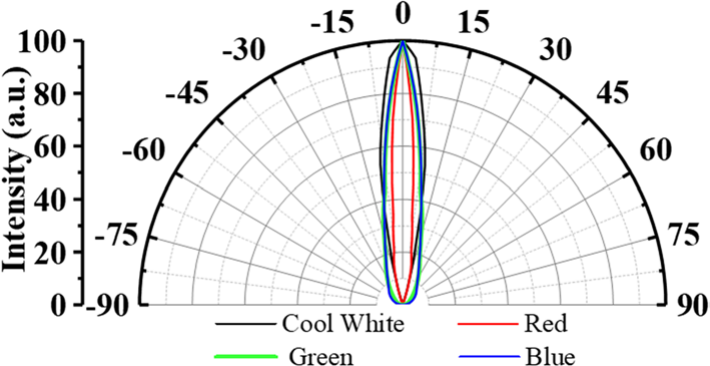
Luminous intensity distribution curves of the four HC mini-LEDs

Direct bilirubin test paper (S-Y U-B Test Kit Lot No. 1110412-SY8076-11011019-Shih-Yung Instruments Co., Ltd.) of diameter 30 mm was used as the analytical direct bilirubin carrier. The four HC mini-LEDs used in this study were provided by EVERLIGHT Corporation, New Taipei, Taiwan. The model, viewing angle, and peak wavelength of the four LEDs are as follows: cool white (334-15/F1C1-1XZA), 10°, 446.8 nm; red (333-2SURC/S400-A8), 8°, 636.1 nm; green (333-2SUGC/S400-A5), 12°, 518.5 nm; blue (333-2SUBC/C470/S400-A6), 8°, 452.9 nm. The luminous intensity distribution curves of the four HC mini-LEDs are shown in Fig. 1.

### Recording images and RGB analysis

Images were captured with an iPhone 8 Plus camera, with the test strip at 90° to the camera lens. The camera’s shutter speed and focal length were 1/15 s and 10 mm, respectively, and the vertical distance between the camera lens and HC mini-LED light source as well as the test strip sample were 10 cm and 15 cm, respectively. P(λ) and R(λ) are the light source distribution and reflection spectra, respectively. After multiplying P(λ) and R(λ), the spectrum of biliverdin on the test paper can be obtained. Finally, the captured image is analyzed using MATLAB software to obtain the grayscale values of the RGB channels. Then, the grayscale values of the RGB channels are divided by the same sampling pixel number, and the average grayscale is used to represent its color composition for analysis.

Converting RGB images to grayscale images is achieved through the transformation of Y, U, and V, where Y represents luminance, U represents chrominance, and V represents chroma. The formula for converting R, G, B to Y, U, V is given as Eq. (2):

2 $$\left( {\begin{array}{*{20}c} Y \\ U \\ V \\ \end{array} } \right) = \left( {\begin{array}{*{20}c} {0.2989} & {0.5870} & {0.1140} \\ { - 0.148} & { - 0.289} & {0.437} \\ {0.615} & { - 0.515} & { - 0.100} \\ \end{array} } \right)\left( {\begin{array}{*{20}c} R \\ G \\ B \\ \end{array} } \right){ }$$

**Fig. 2:**
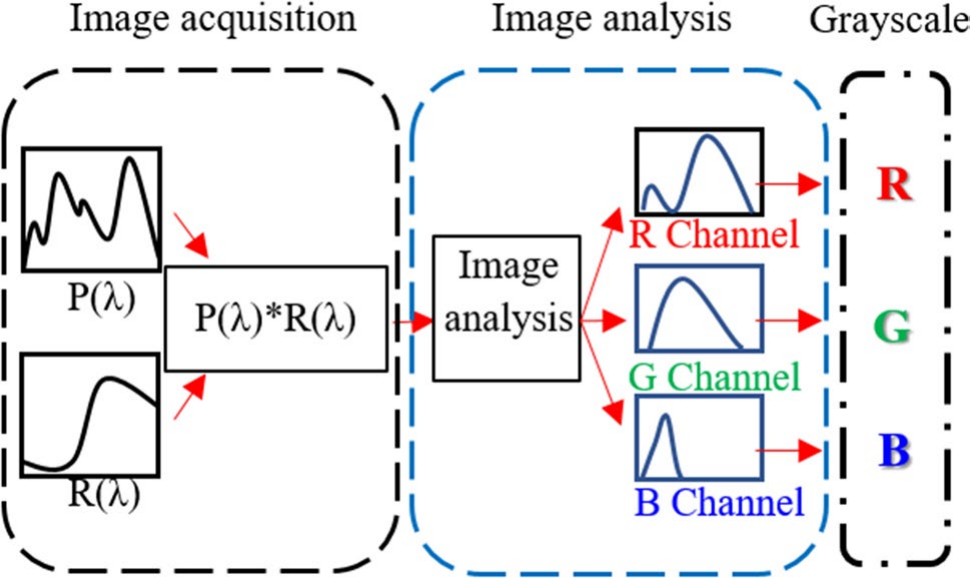
Schematic diagram of the image analysis process

Y = R * 0.2989 + G * 0.5870 + B * 0.1140, which is the grayscale conversion formula [43]. The schematic diagram of the image analysis is shown in Fig. 2.

### Experimental process

In this study, HC mini-LEDs and image grayscale analysis were used to detect direct bilirubin. The HC mini-LEDs were used as light sources, and images were captured with a smartphone. MATLAB software was used to analyze the captured images. Before we start the experimental process, we need to confirm the voltage and current used for the LED, taking into consideration the camera’s ISO sensitivity setting to ensure that the captured images are not overly exposed. Then, process the captured images. We begin by analyzing the grayscale values for the lowest concentration since higher concentration grayscale values will be lower. Therefore, as long as the grayscale values for the lowest concentration do not experience excessive exposure (with values closer to 255 being preferable), it ensures that higher concentrations will also avoid overexposure. Then, the experimental process as below. Step 1 involves pretreatment and zero calibration, where we prepared a total of 12 different concentrations of the direct bilirubin standard solutions from 0.1 to 2.0 mg/dL and added 0.1 mL of Fouchet’s reagent as the oxidant to each solution. Then, the parameters, such as current, voltage, distance of the light source, and focal length of the smartphone, were corrected and fixed. Step 2 involves image processing and image analysis on images captured by the four HC mini-LED light sources. After calibration of the measurement parameters, image acquisition of the 12 direct bilirubin test papers for different concentrations was performed. Step 3 entails finding the best linear R^2^ relationship between the image RGB values. Cool white LEDs were used as the detection light source, and three kinds of monochromatic light HC mini-LEDs were used to analyze the values of the direct bilirubin test papers for different concentrations. The color compositions were represented by the average grayscale values, and the linear relationship between the direct bilirubin concentration and RGB average grayscale was analyzed through R^2^. Step 4 involves comparing the image analysis results captured by the four HC mini-LEDs. The influence of direct bilirubin detection on linearity was compared using different mini-LED light sources. We analyzed the influence of RGB monochromatic and cool white light LEDs on the detection of direct bilirubin.

## Results and discussion

### Light source and experimental architecture

**Fig. 3:**
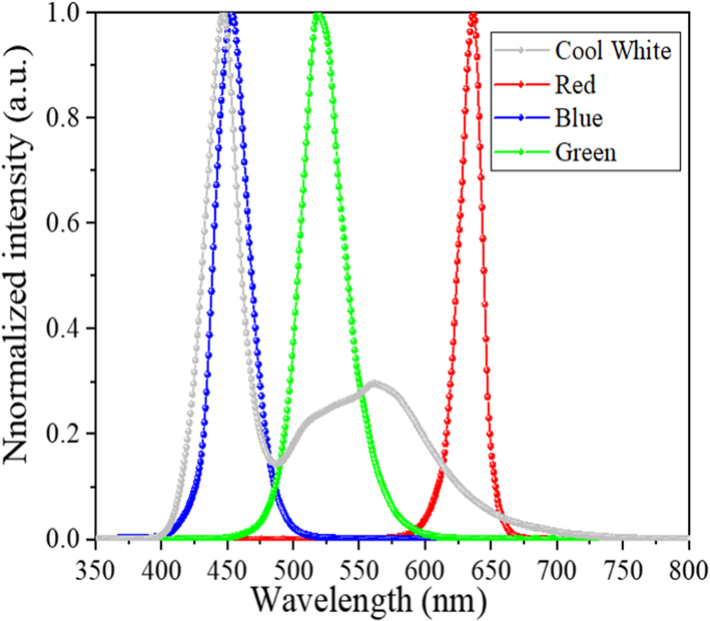
Spectra of the four different HC mini-LEDs

In this study, four HC mini-LEDs were used as detection light sources, and their normalized spectra are shown in Fig. 3.

**Fig. 4:**
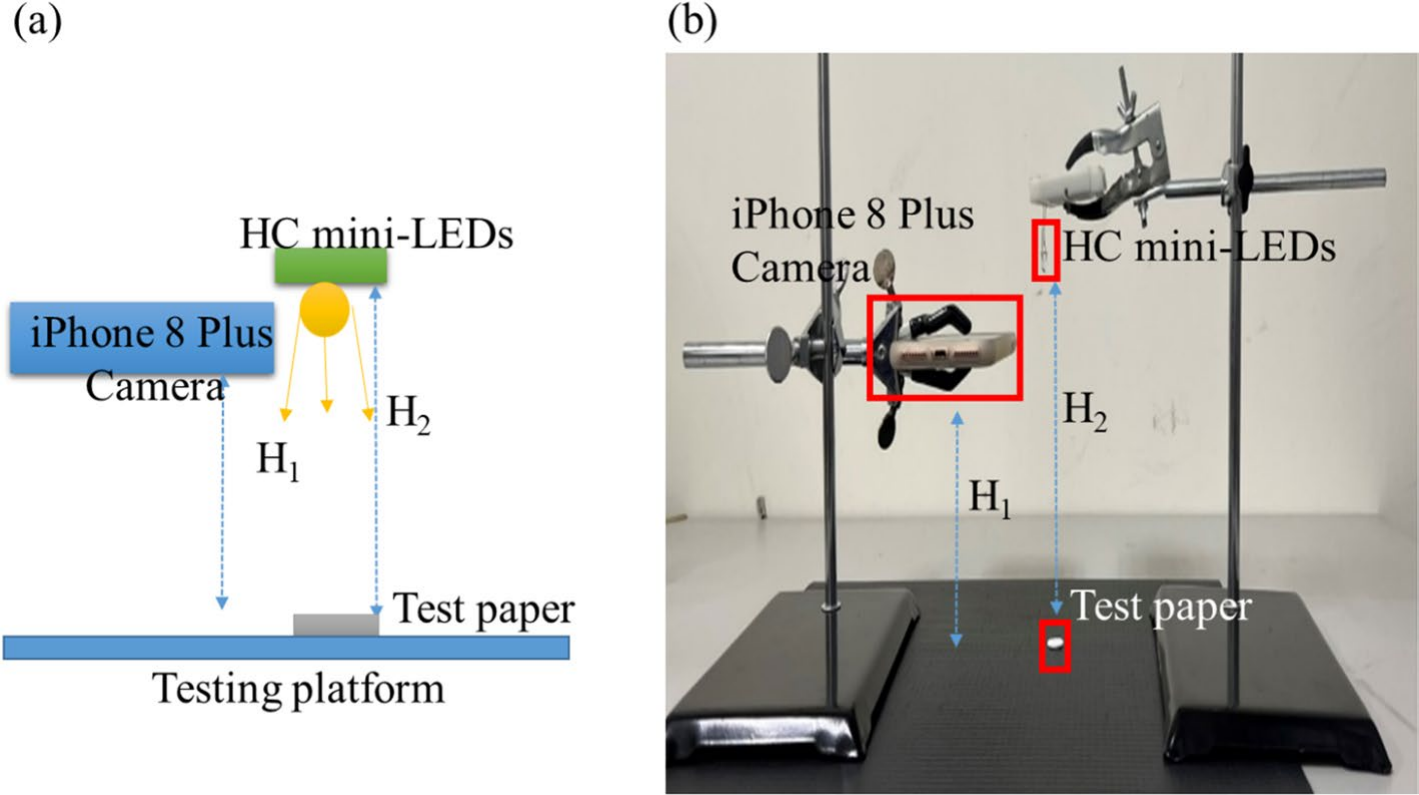
Experimental architecture for direct bilirubin image capture, **a** 2D experimental framework schematic diagram, **b** Actual experimental framework diagram

The experimental architecture for capturing images with an iPhone 8 Plus is shown in Fig. 4. The smartphone and light sources are fixed with brackets, and the test strip and lens are fixed at a 90° angle for image capture. H1 and H2 are the distances from the camera lens to test paper sample and light source to the test paper, respectively. The values of H1 and H2 are 10 cm and 15 cm, respectively.

### Image detection of bilirubin test paper using cool white LEDs as light source

**Fig. 5:**
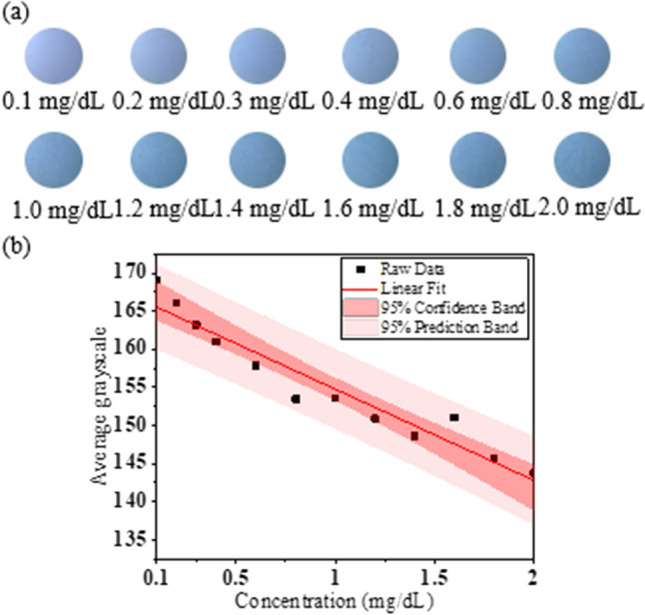
**a** Different concentrations of direct bilirubin test-paper samples irradiated by cool white HC mini-LED. **b** Linear relationship between different concentrations of direct bilirubin and average grayscale in G channel detected by cool white HC mini-LED

In this study, cool white LEDs were used as the light source to detect the direct bilirubin concentration from the test paper. The light source current and voltage were 10 mA and 2.7 V, respectively. Direct bilirubin test papers containing samples with 12 different concentrations ranging from 0.1 to 2.0 mg/dL were studied. In the sample picture shown in Fig. 5a, the camera ISO is set to 25. It is found from the actual sample picture that when the concentration of direct bilirubin is higher, the color of the pattern is darker. This is because higher concentration of bilirubin produces greater proportion of biliverdin, and as the biliverdin deposited on the test paper increases, the color changes. Once the captured images are divided into their R, G, and B channels, MATLAB is used for image processing to analyze the average R, G, and B grayscale values. The average grayscale and direct bilirubin concentration were considered for the linearity analysis. The bilirubin test paper values in the R, G, and B channels corresponded to direct bilirubin R^2^ values of 0.8912 (y = − 21.373x + 142.28), 0.9289(y = − 11.972x + 166.73), and 0.9001 (y =  − 14.932x + 207.43), respectively. The experimental results showed that when using cool white LEDs as the light sources, the best linear relationship was observed for the G channel, where the direct bilirubin concentration range was 0.1–2.0 mg/dL, and the linear equation, R^2^, and LOD for the G channel were y = − 11.972x + 166.73, 0.9289, and 0.567 mg/dL, respectively, as shown in Fig. 5b.

### Image detection of bilirubin test paper using red, green, and blue HC mini-LEDs as light source

**Fig. 6:**
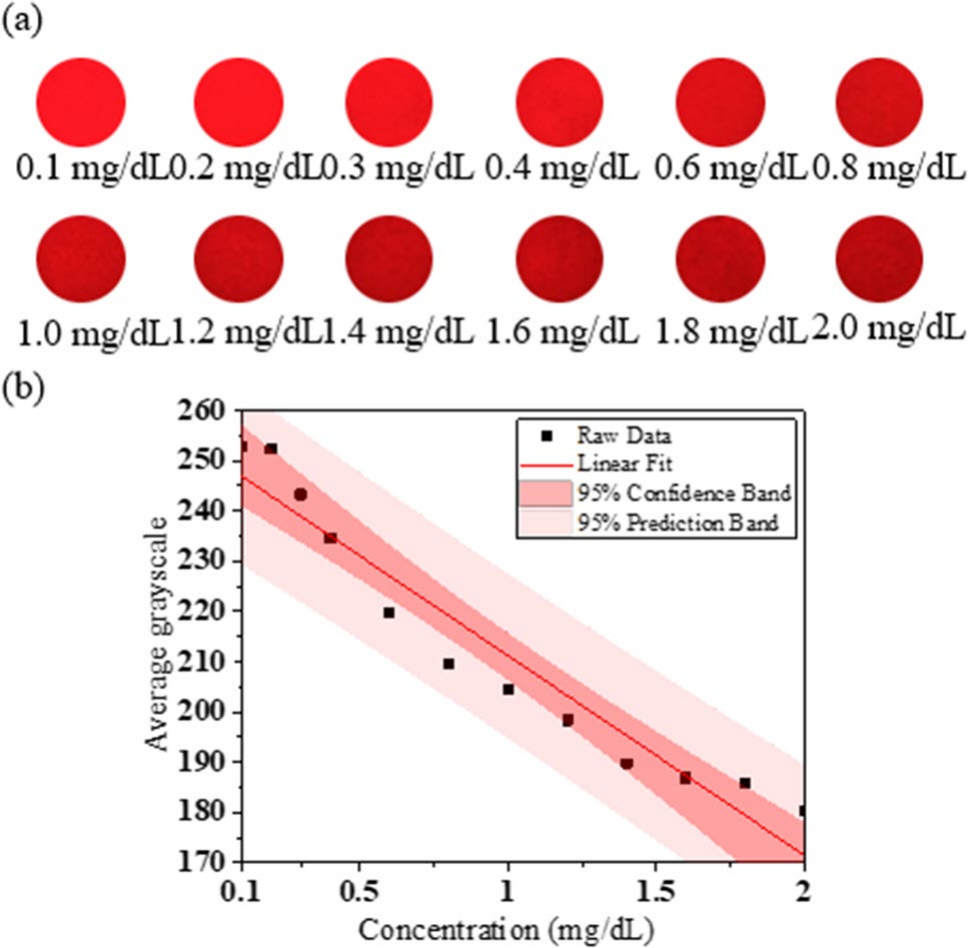
**a** Different concentrations of direct bilirubin test-paper samples irradiated by red HC mini-LED. **b** Linear relationship between different concentrations of direct bilirubin and average grayscale in R channel detected by red HC mini-LED

The current, voltage, and FWHM of the red HC mini-LED were 30 mA, 1.9 V, and 21 nm, respectively. The direct bilirubin test paper results are shown in Fig. 6a, with the camera ISO set to 200. When using the red HC mini-LED, the best linear relationship was in the R channel when the direct bilirubin concentration range was 0.1–2.0 mg/dL, and the linear equation, R^2^, and LOD were y = − 39.708x + 250.92, 0.9365, and 0.534 mg/dL, respectively, as shown in Fig. 6b.

**Fig. 7:**
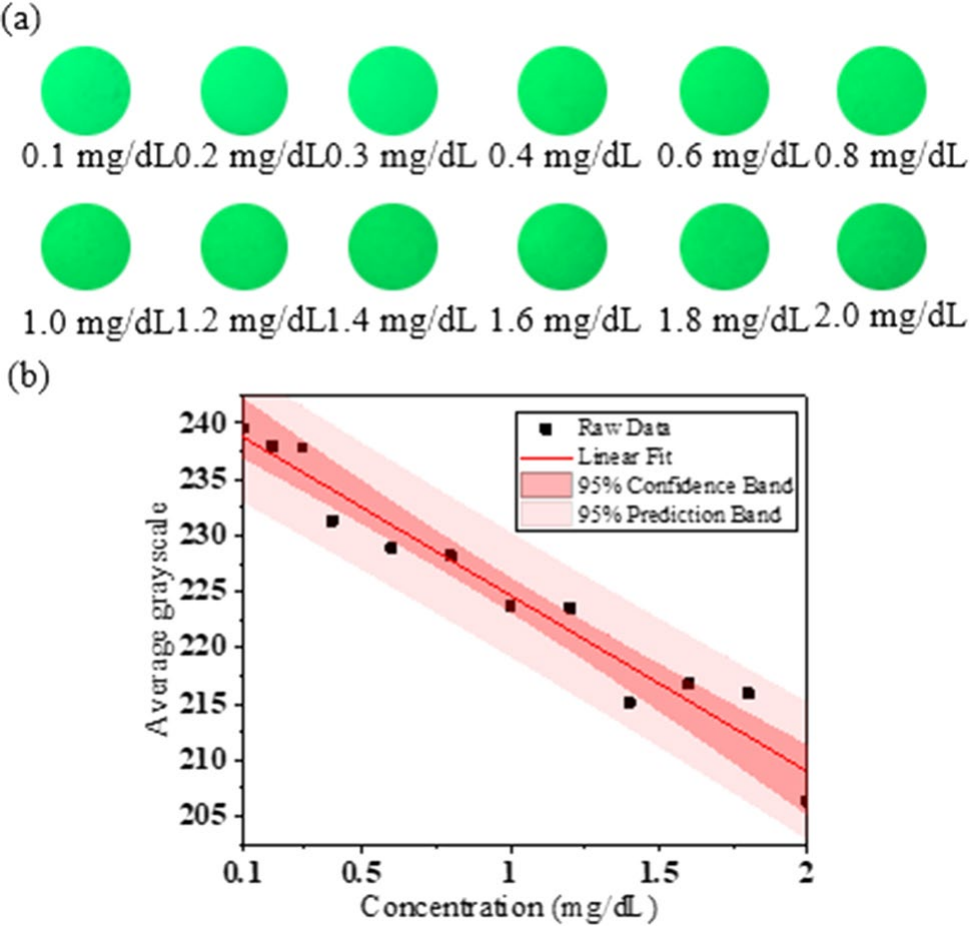
**a** Different concentrations of direct bilirubin test-paper samples irradiated by green HC mini-LED. **b** Linear relationship between different concentrations of direct bilirubin and average grayscale in G channel detected by green HC mini-LED

The current, voltage, and FWHM of the green HC mini-LED were 30 mA, 2.8 V, and 38 nm, respectively. The direct bilirubin test paper results are shown in Fig. 7a, with the camera ISO set to 100. When using the green HC mini-LED, the best linear relationship was in the G channel when the direct bilirubin concentration range was 0.1–2.0 mg/dL, and the linear equation, R^2^, and LOD in the G channel were y = − 15.669x + 240.34, 0.9523, and 0.459 mg/dL, respectively, as shown in Fig. 7b.

**Fig. 8:**
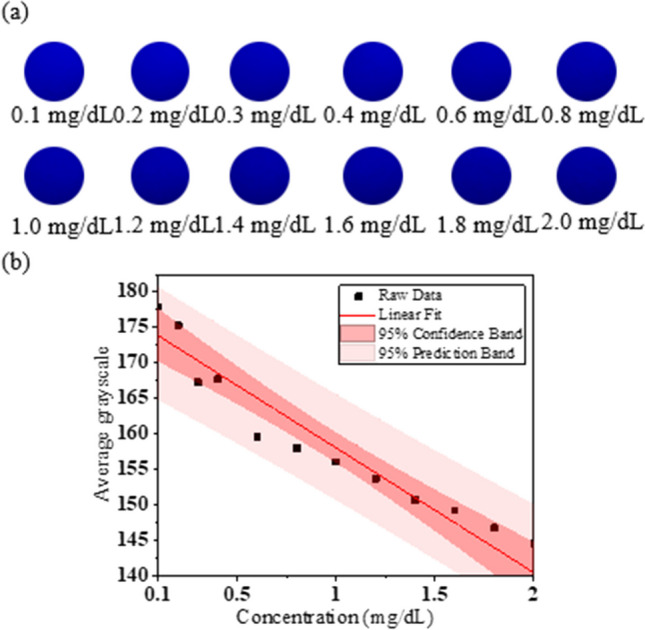
**a** Different concentrations of direct bilirubin test-paper samples irradiated by blue HC mini-LED. **b** Linear relationship between different concentrations of direct bilirubin and average grayscale in B channel detected by blue HC mini-LED

Finally, using the blue HC mini-LED as the light source, the current was 5 mA, voltage was 2.6 V, and FWHM was 30 nm. The direct bilirubin test paper results are shown in Fig. 8a, with the camera ISO set to 25. When using the blue HC mini-LED as the light source, the best linear relationship was in the B channel for a concentration range of direct bilirubin of 0.1–2.0 mg/dL. In the B channel, the linear equation, R^2^, and LOD were y = − 16.253x + 174.29, 0.9224, and 0.595 mg/dL, respectively, as shown in Fig. 8b.

Table 1 shows the specifications of the four types of HC mini-LEDs used and the analytical test paper results. The test paper images were captured with 178,101 pixels, and the RGB average grayscale was taken to analyze the linear relationship. We compared the cool white and monochromatic LEDs for detection of the direct bilirubin test papers in the concentration range of 0.1–2.0 mg/dL. The R^2^ detected by the monochromatic LEDs increased from 0.8912, 0.9289, and 0.9001 to 0.9364, 0.9523, and 0.9224 in the R, G, and B channels, respectively. These results show that the linearity of the bilirubin test paper could be improved when monochromatic light LEDs are used as light sources for image analysis. Moreover, they show that the detection result of the green LED was the best, and its LOD was 0.459 mg/dL. The R^2^ values for Figs. 5, 6, 7 and 8 are 0.9289, 0.9365, 0.9523, and 0.9224, respectively. We analyzed the three RGB channels after converting them to grayscale for analysis. The main source of error in this process is the uniformity of coloration in the test paper. In order to reduce this error, we have also standardized the reaction time and size of the test paper, minimizing the magnitude of this discrepancy as much as possible.

**Table 1:** Comparison of color image analysis results of test papers with different light sources

Light source	Red HC mini-LEDs	Green HC mini-LEDs	Blue HC mini-LEDs	Cool white HC mini-LEDs
Peak wavelength (nm)	636.1	518.5	452.9	446.8
Wavelength range (nm)	575–675	450–625	400–525	380–780
Color coordinates (x, y)	(0.6971,0.3021)	(0.1677, 0.7050)	(0.1496,0.0314)	(0.2345, 0.2067)
The linear equation, R^2^	0.9365	0.9523	0.9224	0.9289 (G channel)
Concentration (mg/dL)	0.1–2			
Analysis points (pixels)	178,101			

## Conclusion

In this study, we propose image spectral analysis with HC mini-LEDs as the light sources for quantitative detection of direct bilirubin. Redox-labeled bilirubin was used to capture images with a smartphone, and image processing was used to perform RGB analysis to achieve quantitative detection of images of the direct bilirubin test paper. The experimental results showed that when green HC mini-LED was used as the light source and the average RGB grayscale of the image was analyzed in the concentration range of 0.1–2.0 mg/dL, the bilirubin concentration and G channel had the best linear relationship. The linear equation, R^2^, and LOD were y = − 15.669x + 240.34, 0.9523, and 0.459 mg/dL, respectively. Moreover, it was possible to quantitatively analyze direct bilirubin concentrations higher than 1.530 mg/dL. The proposed analysis method has the advantages of noninvasiveness, fast detection, and quantitative numerical analysis, and the detected data can be used to more accurately to analyze changes in the patient’s direct bilirubin index so as to provide a reference for hospitals to achieve their telemedicine goals. This study proposes Spectral Analysis with Highly Collimated Mini-LEDs as Light Sources for Quantitative Detection of Direct Bilirubin powder. Real human direct bilirubin detection has not been conducted yet, and future steps involve conducting human trials in collaboration with medical institutions.

## Data Availability

The data presented in this study are available on request from the all authors.

## Abbreviations

Mini-LEDs: Mini-light-emitting diodes
BOD: Bilirubin oxidase
LOD: Limit of detection
CoA: Certificate of analysis
FWHM: Full width at half maximum
R^2^: Correlation of determination
